# Semaglutide Versus Other Glucagon-Like Peptide-1 Agonists for Weight Loss in Type 2 Diabetes Patients: A Systematic Review and Meta-Analysis

**DOI:** 10.7759/cureus.69008

**Published:** 2024-09-09

**Authors:** Jimmy Wen, Denise Nadora, Ethan Bernstein, Christiane How-Volkman, Alina Truong, Muzammil Akhtar, Neha A Prakash, Jose Puglisi, Eldo Frezza

**Affiliations:** 1 Physical Medicine and Rehabilitation, California Northstate University College of Medicine, Elk Grove, USA; 2 Neurology, California Northstate University College of Medicine, Elk Grove, USA; 3 Internal Medicine, California Northstate University College of Medicine, Elk Grove, USA; 4 Cardiology, California Northstate University College of Medicine, Elk Grove, USA; 5 Surgery, California Northstate University College of Medicine, Elk Grove, USA; 6 Medicine, A.T. Still University, Phoenix, USA; 7 Biostatistics, California Northstate University College of Medicine, Elk Grove, USA

**Keywords:** dulaglutide, exenatide, glp-1, liraglutide, semaglutide, tirzepatide, weight loss

## Abstract

Obesity places patients at higher risk for numerous problems, including prediabetes, type 2 diabetes mellitus (T2DM), hypertension, metabolic syndrome, cardiovascular disease, and nonalcoholic fatty liver disease. Glucagon-like peptide-1 receptor agonists (GLP-1 RAs) are antidiabetic drugs that have a recognized effect on weight loss. This systematic review analyzed semaglutide against alternative GLP-1 agonists in facilitating weight loss and evaluated their associated adverse events (AEs) in diabetic patients. A systematic search following the Preferred Reporting Items for Systematic Reviews and Meta-Analyses (PRISMA) guidelines was performed using PubMed, Embase, and Cochrane Library for studies comparing semaglutide and other GLP-1 RAs for weight loss. A narrative synthesis and meta-analysis using SPSS program version 29 were performed to analyze the differences in weight loss between cohorts. Nine studies with 5,445 patients whose mean age was 60.01 years (55.5-70) and mean follow-up of 32.5 weeks (4-58.7) were included. The meta-analysis showed that semaglutide had a greater mean weight loss compared to liraglutide (-6.08, 95% confidence interval (Cl) = -8.40, -3.75) and dulaglutide (-2.85, 95% CI = -5.59, 0.11). Tirzepatide had a greater mean weight loss compared to semaglutide (-3.78, 95% CI = -5.52, -2.04). Common AEs included minor and moderate gastrointestinal events. In conclusion, GLP-1 RAs have shown efficacy in reducing body weight in T2DM patients. Semaglutide, liraglutide, dulaglutide, tirzepatide, and exenatide demonstrated mean weight loss reductions of 4.81 kg, 2.81 kg, 4.03 kg, 9.7 kg, and 1.9 kg, respectively, with high rates of minimal to moderate-severity AEs. Semaglutide demonstrated increased numerical weight loss compared to its comparators (dulaglutide, liraglutide, and exenatide). However, tirzepatide, a dual-agonist, produced greater weight loss compared to semaglutide. The paucity of comparative head-to-head trials prevents a definitive conclusion of the superiority of one GLP-1 RA over another.

## Introduction and background

Obesity, currently defined as a body mass index (BMI) >30 kg/m^2^, places patients at a higher risk for a multitude of problems, such as type 2 diabetes mellitus (T2DM), dyslipidemia, hypertension, cardiovascular disease, osteoarthritis, and multiple forms of cancer [1]. Thus, interventions to combat obesity can have profound short-term and long-term benefits on personal health and welfare. Bariatric surgery is a common surgical weight loss method, while exercise, diet modification, and pharmacologic therapy are common modes of nonsurgical weight loss.

Glucagon-like peptide-1 receptor agonists (GLP-1 RAs) are a class of medications traditionally recognized for their efficacy in controlling hemoglobin A1C levels via insulin secretion augmentation and suppressing glucagon release [2]. GLP-1 RAs cause delayed gastric emptying and stimulation of central receptors involved in appetite suppression and energy expenditure, which has resulted in the recognition of GLP-1 RAs as effective therapy for weight loss in patients with and without T2DM [3]. Several GLP-1 RAs are FDA-approved for the treatment of T2DM. Semaglutide is a GLP-1 RA that has recently been approved for weight loss and has subsequently been growing in popularity.

A comparative analysis rather than indirect comparisons or network analysis reduces the confounding and bias introduced by differences in patient demographics and reported outcomes. Through a comparative analysis between relevant candidates, a more accurate comparison of weight loss and complications can be elucidated. This systematic review aims to assess whether semaglutide, a newer GLP-1 RA, can provide superior weight loss benefits and comparable safety profile compared to other agents in its class. These findings can help guide clinical decision-making and optimize treatment plans for T2DM patients.

## Review

Methodology

Search Strategy

A comprehensive search across three databases, namely, ​​PubMed, Embase, and Cochrane Library, was conducted on November 9, 2023, following guidelines established by the Preferred Reporting Items for Systematic Reviews and Meta-Analyses (PRISMA) guidelines. The following search strategy was utilized across the databases: (semaglutide) AND (exenatide OR liraglutide OR albiglutide OR dulaglutide OR lixisenatide OR tirzepatide OR GLP-1 agonist OR glucagon-like peptide 1 agonist) AND (weight loss). There were no limits set on our search strategies.

Article Selection

A Patient, Intervention, Comparison, and Outcome (PICO) methodology was utilized. The PICO criteria in this study encompassed patients of all ages taking semaglutide or an active comparator GLP-1 RA that evaluated weight loss and safety profile. The following inclusion criteria determined if studies were included for analysis: (1) comparative studies such as randomized controlled trials (RCTs), cohort studies, or case-control studies investigating the effect of semaglutide versus comparator GLP-1 agonists; (2) studies that included post-intervention endpoints such as but not limited to weight loss, BMI, insulin and glucose levels, lipid panel values, and other patient-reported outcomes; and (3) T2DM patients. Exclusion criteria included studies that did not meet all three inclusion criteria and study designs such as noncomparative studies, case reports, review articles, animal studies, cadaveric studies, expert opinions, abstracts, and commentaries. Additionally, studies were excluded if the articles were not in English and if no full text was available. This protocol was registered in the PROSPERO database as CRD42024496591.

All authors contributed to the screening and selection process of articles. Each article was independently reviewed by two reviewers during title/abstract and full-text screening to determine study eligibility. For articles where there was a discrepancy between reviewers, the articles were reviewed by the lead author (JW). A rigorous reference search was performed for all included studies to determine if any additional studies could be added to this review.

Study Quality

The risk of bias and methodological quality were determined utilizing the Methodologic Index for Non-randomized Studies (MINORS) criteria for non-RCTs and the Cochrane Risk of Bias tool for RCTs [4,5]. MINORS scores were reported as 0 (not reported), 1 (reported but inadequate), or 2 (reported and adequate), with a maximum score of 24 for comparative studies (12 categories). The risk of bias was determined as high if the MINORS score was between 0 and 16, moderate if the score was between 16 and 20, and low if the score was between 21 and 24. The Cochrane Risk of Bias tool assesses seven domains, i.e., sequence generation, allocation concealment, blinding of participants/personnel, blinding of outcomes, incomplete outcome data, selective outcome reporting, and other sources of bias. These criteria were evaluated as high, low, or unclear risk of bias. Two independent authors assessed the risk of bias and methodological quality and any disagreements were resolved via rigorous re-review or by consulting a third reviewer until consensus was achieved.

Data Extraction and Analysis

Study variables included within this systematic review included title, author, publication date, study year, number of patients, dosage of drug, mean age, mean follow-up time, BMI, laboratory values (insulin, glucose, lipid panel (triglycerides, low-density lipoprotein, high-density lipoprotein), and patient-reported outcomes. The data extraction database was compiled utilizing Microsoft Excel (Microsoft Office Version 16.80 2023). SPSS version 29 (IBM Corp., Armonk, NY, USA) was used to perform a meta-analysis via a random-effects model to compare mean weight loss numbers between semaglutide versus other GLP-1 RAs included in this study. Heterogeneity was calculated using Cochran’s Q, Higgins’ I-squared, and Tau squared. Forest plots were created using GraphPad Prism version 10.

Results

Study Selection and Characteristics

**Figure 1:**
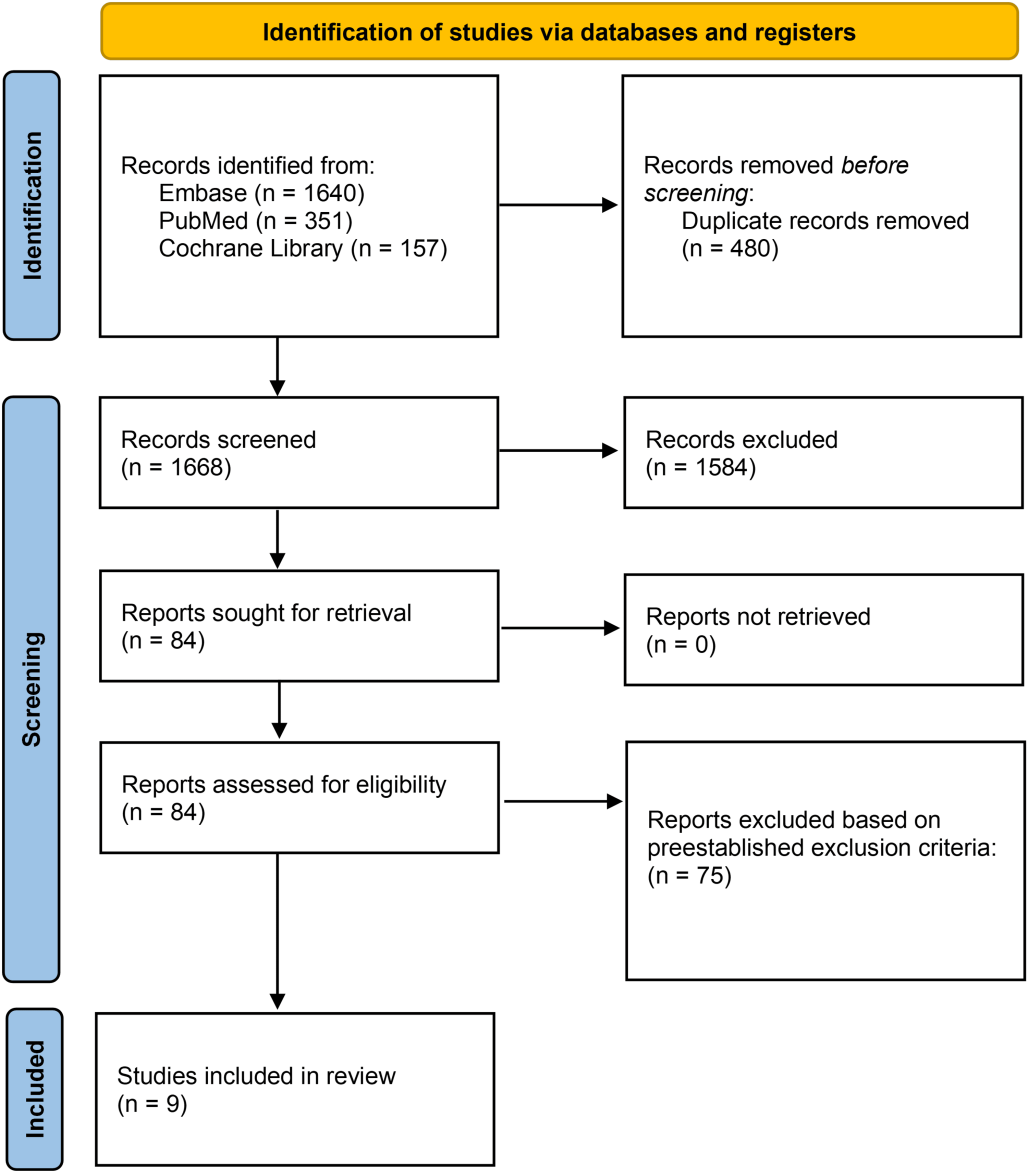
Preferred Reporting Items for Systematic Reviews and Meta-Analyses (PRISMA) flow diagram of the article selection process.

The initial search yielded a total of 2,148 studies from Cochrane Library, Embase, and PubMed. A total of 480 duplicate articles were removed and the remaining 1,668 articles were screened based on their title and abstracts, leaving 84 to be reviewed. A thorough full-text review was conducted and yielded nine studies to be included in this systematic review. The screening process can be visualized in Figure 1.

Across all nine studies, there were a total of 5,445 patients, with a total of 5,183 patients who completed the studies [6-14]. The mean age was 60.01 years, with the mean ages ranging between 55.5 and 70 years (Table 1).

**Table 1:** Patient demographics. SD: standard deviation

Author	Number of patients enrolled	Number of patients who completed the study	Sex (female, male)	Mean age in years (SD)
Capehorn et al., 2019 [9]	577	569	250, 327	59.5 (10.2)
Ahmann et al., 2018 [8]	809	743	362, 447	56.6 (20-83)
Frías et al., 2021 [6]	1,878	1,783	996, 882	56.6 (10.4)
Heise et al., 2022 [7]	117	108	31, 86	61.9
Iijima et al., 2023 [10]	32	30	6, 26	62.1 (11.5)
Pratley et al., 2019 [12]	711	685	341, 370	56 (10)
Pratley et al., 2018 [11]	1,201	1,129	537, 662	Semaglutide 0.5 mg: 56; semaglutide 1.0 mg: 55; dulaglutide 0.75 mg: 55; dulaglutide 1.5 mg: 56, 55.5
Seijas-Amigo et al., 2023 [13]	94	90	43, 51	61.9 (10.9)
Thomas et al., 2023 [14]	46	46	2, 44	70

Following the screening process, the systematic review encompassed seven RCTs, one nonrandom observational study, and one retrospective cohort study, comparing semaglutide with several GLP-1 RAs such as liraglutide, exenatide, tirzepatide, and dulaglutide. Among these trials, two investigated the efficacy of a 1.0 mg weekly dosage of semaglutide compared to tirzepatide [6,7]. In a third trial, the comparison involved 1.0 mg of semaglutide versus 2.0 mg once weekly of exenatide [8]. Additionally, a fourth trial compared 1.0 mg of semaglutide to a daily dose of 1.2 mg of liraglutide [9]. The remaining trials examined different dosages of semaglutide against varying doses of different GLP-1 RAs [10-14].

For instance, one trial compared patients who received semaglutide at 0.25 mg once weekly, then escalated to 0.5 mg once weekly, against patients who received 0.75 mg of dulaglutide once weekly [10]. Another trial compared patients who received a dosage regimen of 14 mg of semaglutide against 1.8 mg of liraglutide [12]. Additionally, a third trial compared patients receiving either 0.5 mg or 1.0 mg of semaglutide once weekly against those receiving 0.75 mg or 1.5 mg of dulaglutide once weekly [11]. The last two trials did not specify the dosages of the GLP-1 RAs [13,14].

In the study by Seijas-Amigo et al., patients were compared based on the route of administration, with one group receiving semaglutide subcutaneously and orally, and the other group receiving dulaglutide subcutaneously [13]. The retrospective cohort study by Thomas et al., on the other hand, evaluated patients receiving semaglutide, liraglutide, or dulaglutide subcutaneously [14].

Two of the studies included placebo control groups. The mean follow-up was 32.5 weeks, with the mean follow-up ranging between four and 58.7 weeks (Table 2).

**Table 2:** Study characteristics. SQ: subcutaneous

Author	Number of patients assigned to semaglutide	Semaglutide intervention	Number of patients assigned to comparator	Comparator intervention	Number of patients assigned to placebo	Mean follow-up (weeks)
Capehorn et al., 2019 [9]	290 (287 completed)	1.0 mg SQ once weekly for 30 weeks (8-week dose escalation). Subjects started at 0.25 mg and escalated in 4-week increments until 1.0 mg was reached	Liraglutide 287 (282 completed)	1.2 mg SQ daily for 30 weeks with a 1-week dose escalation period. Subjects started at 0.6 mg for 1 week and escalated to 1.2 mg	0	30
Ahmann et al., 2018 [8]	404	1.0 mg SQ once weekly for 56 weeks. Subjects started at 0.25 mg and escalated in 4-week increments until 1.0 mg was reached	Exenatide 405	2.0 mg SQ once weekly for 56 weeks	0	5
Frías et al., 2021 [6]	469	1 mg once weekly for 40 weeks. Subjects started at 0.25 mg and escalated in 4-week increments until 1.0 mg was reached	Tirzepatide 5 mg: 470; 10 mg: 469; 15 mg: 470	Once weekly SQ for 40 weeks. Subjects started at 2.5 mg and escalated by 2.5 mg every 4 weeks until the assigned dose was reached.	0	4
Heise et al., 2022 [7]	44	1 mg once weekly for 20 weeks. Subjects started at 0.25 mg and escalated in 4-week increments until 1.0 mg was reached	Tirzepatide 45	15 mg (2·5 mg, 5·0 mg, 7·5 mg, 10·0 mg, and 12·5 mg for 4 weeks each, followed by 15 mg for the remaining 8 weeks)	28	28
Iijima et al., 2023 [10]	16	0.25 mg for 4 weeks followed by 0.5 mg once weekly	Dulaglutide: 16	0.75 mg of dulaglutide once weekly	0	26
Pratley et al., 2019 [12]	286	Oral semaglutide was initiated once daily treatment at 3 mg with dose escalation to 7 mg at 4 weeks and to the maintenance dose of 14 mg at 8 weeks	Liraglutide: 284	Liraglutide: SQ liraglutide initiated treatment at 0·6 mg once daily with dose escalation to 1.2 mg after 1 week and to the maintenance dose of 1·8 mg after 2 weeks	142	52
Pratley et al., 2018 [11]	0.5 mg: 301; 1.0 mg: 300	0.5 mg/1.0 mg once a week SQ	Dulaglutide: 0.75 mg: 299; 1.5 mg: 299	0.75 mg/ 1.5 mg once a week SQ	-	40
Seijas-Amigo et al., 2023 [13]	SQ Sema: 40; Oral: 28	SQ vs. oral	Dulaglutide: 21	SQ	-	13
Thomas et al., 2023 [14]	20	SQ	Liraglutide: 8; dulaglutide: 36	SQ	-	58.7

Risk of Bias Assessment

To assess the qualities of the studies as well as the risk of biases present within the studies, the MINORS and Cochrane Risk of Bias criteria were used given the inclusion of both RCT and non-RCT studies. The included studies had a MINORS score that ranged between 15 and 24. The overall risk of bias was low in six studies, medium in two studies, and high in one study (Table 3). The Cochrane Risk of Bias tool was also used to assess bias in RCTs. The sequence generation showed a low risk of bias across five studies with two studies demonstrating an unclear bias. The allocation concealment showed varying risks of bias. The blinding of participants and personnel as well as the blinding of outcome assessors showed a high risk of bias for five studies and a low risk of bias for two studies, respectively. The risk of bias was low for both the incomplete outcome data and the selective outcome reporting. In terms of other sources of bias, six studies showed a low risk of bias, while one study was identified to have a high risk of bias (Table 4).

**Table 3:** Methodological Index for Non-randomized Studies (MINORS).

Author	Clearly stated aim	Inclusion of consecutive patients	Prospective data collection	Endpoints appropriate to study aim	Unbiased assessment of study endpoint	Follow-up period appropriate to study aim	Loss to follow-up less than 5%	Prospective calculation of study size	Adequate control group	Contemporary groups	Baseline equivalence of groups	Adequate statistical analyses	Total score
Capehorn et al., 2019 [9]	2	2	2	2	0	1	2	2	1	2	2	2	21/24
Ahmann et al., 2018 [8]	2	2	2	2	2	1	0	2	1	2	2	2	20/24
Frías et al., 2021 [6]	2	2	2	2	2	2	0	2	1	2	2	2	21/24
Heise et al., 2022 [7]	2	2	2	2	2	2	0	2	2	2	2	2	22/24
Iijima et al., 2023 [10]	2	2	2	2	0	1	0	2	0	2	0	2	15/24
Pratley et al., 2019 [12]	2	2	2	2	2	2	2	2	2	2	2	2	24/24
Pratley et al., 2018 [11]	2	2	2	2	2	2	2	2	2	2	1	2	23/24
Seijas-Amigo et al., 2023 [13]	2	2	2	2	0	2	1	0	1	2	1	2	17/24
Thomas et al., 2023 [14]	2	2	2	2	0	2	2	0	1	0	1	1	15/24

**Table 4:** Cochrane risk of bias.

Author	Sequence generation	Allocation concealment	Blinding of participants and personnel	Blinding of outcome assessors	Incomplete outcome data	Selective outcome reporting	Other sources of bias
Capehorn et al., 2019 [9]	Unsure	Unsure	High	High	Low	Low	Low
Ahmann et al., 2018 [8]	Low	High	High	High	Low	Low	High
Frías et al., 2021 [6]	Low	Unsure	High	High	Low	Low	Low
Heise et al., 2022 [7]	Low	Low	Low	Low	Low	Low	Low
Iijima et al., 2023 [10]	Unsure	High	High	High	Low	Low	Low
Pratley et al., 2019 [12]	Low	Low	Low	Low	Low	Low	Low
Pratley et al., 2018 [11]	Low	Unsure	High	High	Low	Low	Low

Weight Loss Outcomes

**Figure 2:**
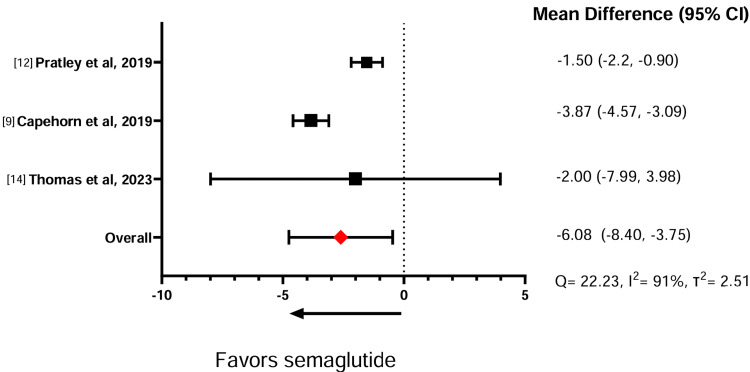
Effect of semaglutide on body weight compared to liraglutide.

**Figure 3:**
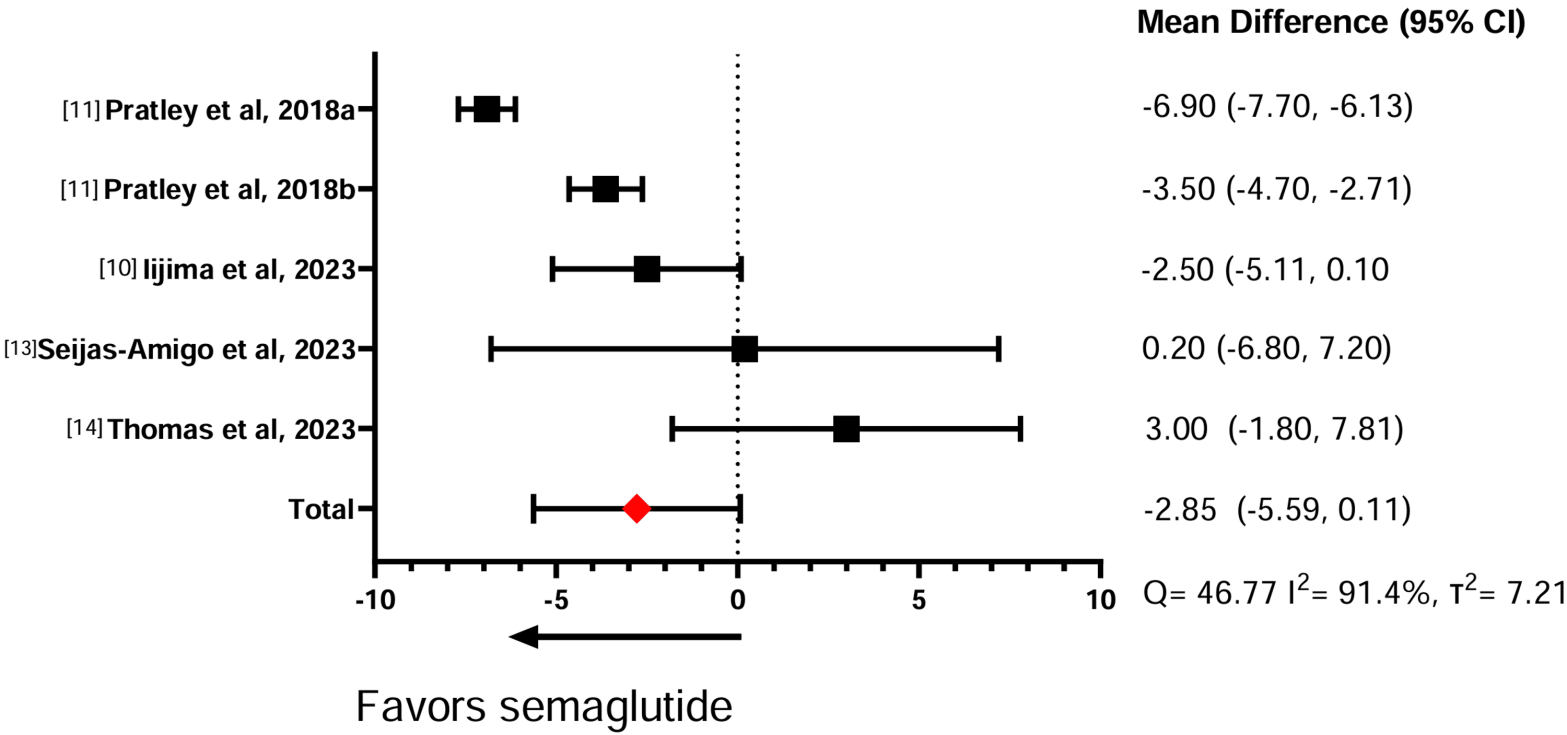
Effect of semaglutide on body weight compared to dulaglutide. a: semaglutide (1 mg) vs. dulaglutide (1.5 mg); b: semaglutide (0.5 mg) vs. dulaglutide (0.75 mg).

**Figure 4:**
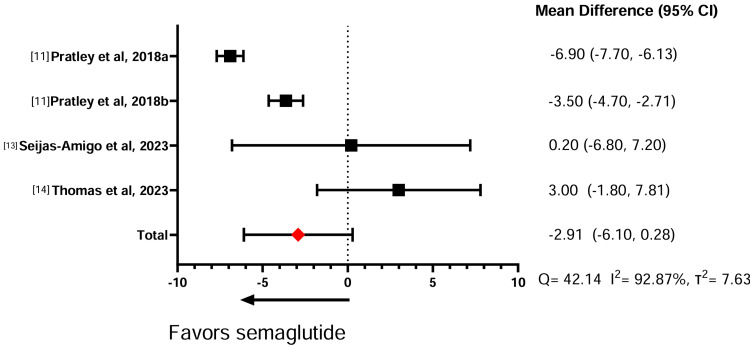
Sensitivity analysis of semaglutide compared to dulaglutide weight loss effects.

**Figure 5:**
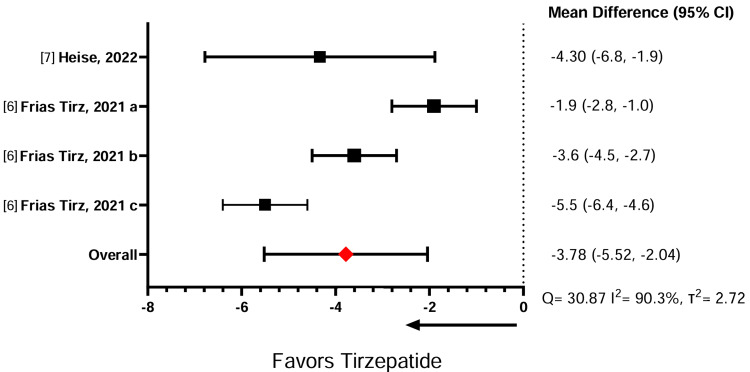
Effect of semaglutide on body weight compared to tirzepatide. a: tirzepatide 5 mg; b: tirzepatide 10 mg; c: tirzepatide 15 mg.

Table 5 illustrates the effect of the GLP-1 RAs on weight change. In three comparative studies between semaglutide against liraglutide, the meta-analysis showed that semaglutide induces significantly more weight loss than liraglutide (standardized mean difference (SMD) = -6.08, 95% confidence interval (CI) = -8.40, -3.75) (Figure 2). Similarly, across four studies comparing semaglutide with dulaglutide, the meta-analysis showed that semaglutide had a significantly greater effect in promoting weight loss compared to dulaglutide (SMD = -2.85, 95% CI = -5.59, 0.11) (Figure 3). A sensitivity analysis was conducted without Lijima et al. given its high risk of bias, and no significant changes were noted for weight loss or heterogeneity calculations (SMD = -2.91, 95% CI = -6.10,0.28) (Figure 4). Finally, in two studies comparing semaglutide against tirzepatide, the meta-analysis showed that tirzepatide showed a significantly greater effect in promoting weight loss compared to semaglutide (SMD = -3.78, 95% CI = -5.52, -2.04) (Figure 5). The study by Frias et al. compared semaglutide against varying doses of tirzepatide. A meta-analysis could not be performed between semaglutide and exenatide due to insufficient data.

**Table 5:** Effect of semaglutide on body weight compared to other GLP-1 agonists. Comparator is defined as an active comparator with another GLP-1 agonist, placebo controls are not included in the table. GLP-1: glucagon-like peptide-1; SQ: subcutaneous; SD: standard deviation

Author	Pre-semaglutide intervention, kg (SD)	Post-semaglutide intervention, kg (SD)	% change (SD)	Pre-comparator intervention, kg (SD)	Post-comparator intervention, kg (SD)	% change (SD)
Capehorn et al., 2019 [9]	96.6 (21)	91 (4.7)	-5.8	Liraglutide: 97.2	Liraglutide 95.26 (4.1)	-2
Ahmann et al., 2018 [8]	96.2	90.6 (0.29)	-5.82	Exenatide: 95.4	Exenatide: 93.59 (0.29)	-1.9
Frías et al., 2021 [6]	93.7 (21.12)	88	-6.08	Tirzepatide: 5 mg: 92.5 (21.76)	Tirzepatide: 84.9	5 mg: -8.22
				10 mg: 94.8 (22.71)	85.5	10 mg: -9.8
				15 mg: 93.8 (21.83)	82.6	15 mg: -11.9
Heise et al., 2022 [7]	92.2 (2.2)	87.7 (0.9)	-4.88	Tirzepatide: 94.1 (2.1)	Tirzepatide: 83.4 (0.9)	-11.4
Iijima et al., 2023 [10]	72.3 (20.2)	70.8 (21.5)	-2.07	Dulaglutide: 72.7 (14.9)	Dulaglutide: 74.3 (14.5)	2.15
Pratley et al., 2019 [12]	92.9 (20.6)	Treatment policy estimand: week 26: 88.5; week 52: 88.6. Trial product estimand: week 26: 88.2; week 52: 87.9	Treatment policy estimand: week 26: -4.74; week 52: -4.63. Trial product estimand: week 26: -5.06; week 52: -5.38	Liraglutide: 95.5 (21.9); placebo: 93.2 (20.0)	Liraglutide treatment policy estimand: week 26: 92.4; week 52: 92.5. Trial product estimand: week 26: 92.3; week 52: 92.4. Placebo: treatment policy estimand: week 26: 92.7; week 52: 92.2. Trial product estimand: week 26: 92.5; week 52: 92	Liraglutide treatment policy estimand: week 26: -3.25; week 52: -3.14. Trial product estimand: week 26: -3.35; week 52: -3.25. Placebo: treatment policy estimand: week 26: -0.54; week 52: -1.07. Trial product estimand: week 26: -0.75; week 52: -2.29
Pratley et al., 2018 [11]	0.5 mg: 96.4 (24.4)	0.5 mg: 91.8	-4.77	Dulaglutide: 0.75 mg: 95.6 (23.0)	Dulaglutide: 0.75 mg: 93.3 (0.27)	-2.41
	1 mg: 95.5 (20.9)	1 mg: 89 (0.28)	-6.81	1.5 mg: 93.4 (21.8)	1.5 mg: 90.4 (0.27)	-3.21
Seijas-Amigo et al., 2023 [13]	99.3 (19.2)	SQ: 94.6; oral: 94.4	SQ: -4.73; oral: -4.93	SQ dulaglutide: 99.3 (19.2)	SQ dulaglutide: 94.3	-5.04
Thomas et al., 2023 [14]	101	96 (8)	-4.95	Liraglutide: 101; dulaglutide: 101	Liraglutide: 98 (7); dulaglutide: 93 kg (9)	Liraglutide: -2.97; dulaglutide: -7.92

Factors Associated with Weight Loss

All patients included in this systematic review were adults who had long-standing type 2 diabetes and were on stable antihyperglycemic medicine with unchanged dosages before screening and the study period. Iijima et al. included patients who were previously treated with 0.6 mg or 0.9 mg of liraglutide and then were switched to semaglutide or dulaglutide. There was a significant decrease in body weight for patients who switched to semaglutide (p = 0.0153) compared to patients who switched to dulaglutide (p = 0.8432). Body weight between the two groups was significantly different (p = 0.0469). Further analysis confirmed differences in the effects of semaglutide and dulaglutide among Japanese patients. It was clinically determined that 0.75 mg of dulaglutide does not promote changes in body weight in Japanese patients [10].

Similarly, the study by Seijas-Amigo et al. included patients who were previously prescribed GLP-1 RAs (semaglutide, liraglutide, exenatide, dulaglutide, or lixisenatide). In this study, the majority of the patients had various significant past medical histories, including pancreatitis, dyslipidemia, and hypertension. This prospective study examined patients who were administered GLP-1 RAs during the initial titration phase. However, it is important to note that 20% of the sample did not reach the maximum dose of the medication, despite tolerating it well. Possible reasons mentioned included the saturation of the primary healthcare system, which may have prevented patients from receiving their monthly follow-ups. The study also showed that 56% of patients who reached the maximum dose achieved weight loss greater than 5%, whereas only 37% of those on medium doses failed to achieve this level of weight loss [13]. Furthermore, in a study in 2019 by Pratley et al., patients who received oral semaglutide with doses escalated to 14 mg compared to patients who received subcutaneous liraglutide. Those estimands were defined as the treatment policy and the trial product. Under both estimands, oral semaglutide was more effective at promoting weight loss compared to both liraglutide over 26 weeks with a weight loss difference of 1.2 kg and 1.5 kg, respectively [12].

Thomas et al. investigated the effectiveness of semaglutide, liraglutide, and dulaglutide in patients with T2DM and end-stage renal disease or undergoing hemodialysis, focusing primarily on changes in hemoglobin A1C levels. The results revealed a statistically significant average weight loss of 6 kg across all patients in the study. Among these patients, 78% experienced a decrease in weight while undergoing GLP-1 RA therapy. Within this group, the average weight loss was 9 kg, with 80% of patients losing more than 5 kg. However, the study did not provide evidence indicating the superiority of one particular GLP-1 RA over another [14].

Adverse Events

AEs were observed in a wide range of participants, ranging from 32% to 98% of the study population. The most frequent AEs reported were gastrointestinal (GI) issues, such as nausea, constipation, vomiting, and diarrhea. Among the studies that provided data on the percentage of participants discontinuing treatment due to AEs, this ranged from 0% to 9%. A study conducted by Seijas-Amigo et al. only presented overall AEs and total GI events without noting specific AEs. The AEs are presented in Table 6.

**Table 6:** Common adverse events with an emphasis on gastrointestinal events. AE: adverse events; SQ: subcutaneous

	Semaglutide								Active comparator							
	Capehorn et al., 2019 [9]	Ahmann et al., 2018 [8]	Frías et al., 2021 [6]	Heise et al., 2022 [7]	Iijima et al., 2023 [10]	Pratley 2019 [12]	Pratley et al., 2018 [11]	Seijas-Amigo et al., 2023 [13]	Capehorn et al., 2019 [9]	Ahmann et al., 2018 [8]	Frías et al., 2021 [6]	Heise et al., 2022 [7]	Iijima et al., 2023 [10]	Pratley et al., 2019 [12]	Pratley et al., 2018 [11]	Seijas-Amigo et al., 2023 [13]
Total adverse events	70.60%	75%	64.20%	98%		80%	0.5 mg: 68%; 1 mg: 69%	Oral 36%; SQ: 32%	Liraglutide: 66.2%	76.30%	5 mg: 63.6%; 10 mg: 68.7%; 15 mg: 68.9%	Tirzepatide: 96%; placebo: 79%		Liraglutide: 74%; placebo: 67%	0.75 mg: 62%; 1.5 mg: 74%	76%
Gastrointestinal	43.9		41.20%		12%		0.5 mg: 43%; 1.0 mg: 44%	SQ: 22%; oral: 25%	38.30%		5 mg: 40%; 10 mg: 46.1%; 15 mg: 44.9%		3%		Dulaglutide 0.75 mg: 1.5 mg:	62%
Nausea	21.80%	22.30%	17.90%	30%	4%	20%	0.5 mg: 23%; 1.0 mg: 21%		15.70%	11.90%	5 mg: 17.4%; 10 mg: 19.2%; 15 mg: 22.1%	Tirzepatide: 24%; placebo: 25%	0%	Liraglutide: 18%; placebo: 4%	0.5 mg: 13%; 1.0 mg: 20%	
Obstipation/Constipation	5.90%	6.40%	5.80%	18%	4%	8%	0.5 mg: 5%; 1.0 mg: 5%		3.60%	5.20%	5 mg: 6.8%; 10 mg: 4.5%; 15 mg: 4.5%	Tirzepatide: 13%; placebo: 0%	2%	Liraglutide: 4%; placebo: 4%	0.5 mg: 3%; 1.0 mg: 5%	
Vomiting	10.40%	7.20%	8.30%	11%	2%	9%	0.5 mg: 10%; 1.0 mg: 10%		8.00%	6.20%	5 mg: 5.7%; 10 mg: 8.5%; 15: 9.8%	Tirzepatide: 7%; placebo: 4%	0%	Liraglutide: 5%; placebo: 2%	0.5 mg: 4%; 1.0 mg: 10%	
Diarrhea	15.60%	11.40%	11.50%	30%	4%	15%	0.5 mg: 14%; 1.0 mg: 14%		12.20%	8.40%	5 mg: 13.2%; 10 mg: 16.4%; 15 mg: 13.8%	Tirzepatide: 20%; placebo: 21%	1%	Liraglutide: 11%; placebo: 8%	0.5 mg: 8%; 1.0 mg: 18%	
Headache		9.40%		16%		9%	0.5 mg: 8%; 1.0 mg: 7%			9.60%		Tirzepatide: 9%; placebo: 18%		Liraglutide: 6%; placebo: 6%	0.5 mg: 4%’ 1.0 mg: 6%	
Discontinuations due to AE	11%	9.40%	4.10%	0	1%	11%	0.5 mg: 8%; 1.0 mg: 10%		6.60%	7.20%	5 mg: 6.0%; 10 mg: 8.5%; 15 mg: 8.5%	Tirzepatide: 2%; placebo: 11%	0%	Liraglutide: 9%; placebo: 4%	0.5 mg: 5%; 1.0 mg: 7%	

Discussion

This systematic review analyzed nine studies (seven RCTs/two non-RCTs) evaluating weight loss in type 2 diabetic patients treated with semaglutide versus another GLP-1 RA (liraglutide, dulaglutide, tirzepatide, exenatide). Three studies compared semaglutide with liraglutide, four studies compared semaglutide with dulaglutide, two studies compared semaglutide with tirzepatide, and one study compared semaglutide with exenatide. The main findings of this study were that semaglutide provided greater weight loss compared to all the comparator GLP-1 RAs except for tirzepatide. All GLP-1 RAs demonstrated substantial GI AEs.

Obesity is associated with several comorbidities such as cardiovascular disease (e.g., hypertension, heart failure), endocrine alterations (e.g., diabetes, metabolic disease), respiratory issues (e.g., apnea, arrhythmia), several cancers, and overall reduced quality of life [15]. The increase in the prevalence of obesity has been concomitantly linked with an increase in the prevalence of T2DM [16]. Therefore, reductions in body weight can be an effective ameliorating factor for these comorbidities and is an important component of diabetes management. Diabetes prevalence has increased drastically over the last couple of decades and was estimated to be at 10.5% and is projected to rise to 12.2% in 2045 [17]. Weight loss between 5% and 7% has been associated with improved metabolic control and reduced comorbidities of diabetes, such as cardiovascular disease [13]. Additionally, weight loss of 15% has been shown to lead to remission of T2DM as well albeit after bariatric surgery. Therefore, pharmacological methodologies may become an attractive alternative without the invasiveness of surgical procedures [18,19].

A post-hoc analysis of the SUSTAIN trials showed that BMI levels did not influence the significance of weight loss compared to placebo and their active comparators, including sitagliptin, exenatide, dulaglutide, and insulin glargine. This significance was also observed for semaglutide patients who achieved ≥5% and ≥10% weight loss (p < 0.05 for all) [20]. Similarly, liraglutide, exenatide, and dulaglutide exhibited weight loss regardless of BMI subgroups [21-23].

Comparative Efficacy

Before the development of semaglutide, liraglutide, in direct comparison to other GLP-1 RAs (exenatide, albiglutide, dulaglutide, lixisenatide), showed significantly greater weight loss [20,24]. A 2021 network meta-analysis found a significant dose-dependent weight loss of semaglutide 0.5 and 1 mg compared to 0.6 mg liraglutide, but not in comparison to 1.2 and 1.8 mg liraglutide [25]. More recently, a 2024 network meta-analysis found significant weight loss across all GLP-1 RAs compared to placebo, with liraglutide, dulaglutide, exenatide, semaglutide, and tirzepatide producing mean differences of -1.33 kg, -0.73 kg, -0.62 kg, -3.13 kg, and -8.47 kg, respectively [26]. Similarly, in this study, semaglutide produced a greater numerical weight loss compared to the other GLP-1 RA active comparators except for tirzepatide. Tirzepatide showed a significant reduction in weight loss compared to semaglutide which may be attributed to its ability to act as a dual agonist of GLP-1 and gastric inhibitory polypeptide (GIP) receptors. Tirzepatide’s advantage is via its dual agonism decreasing hyperglycemia and appetite significantly more than other GLP-1 RAs, possibly leading to its improved reductions in body weight compared to semaglutide [27]. The long-acting GLP-1 RAs tend to produce more significant weight reduction compared to the short-acting variants [27].

GLP-1 RA’s weight loss has been attributed to their ability to decrease gastric emptying, suppress appetite, increase satiety, and exhibit glucose-dependent insulin secretion which contributes to improved glycemic control and subsequent weight loss in diabetic patients [26]. Additionally, there appears to be a centrally mediated effect as well through direct interaction with the hypothalamus and activation of proopiomelanocortin/cocaine-and-amphetamine-regulated transcript, as well as the inhibition of neuropeptide Y and agouti-related peptide [20,28]. Additionally, a 12-week RCT between semaglutide and placebo found a lower caloric intake during lunch, dinner, and with snacks (p < 0.0001, p = 0.0401, and p = 0.0034, respectively), with an overall reduction of caloric intake across all meals throughout the day (p < 0.0001) [29]. These findings were attributed to decreased hunger and food cravings as well as a lower preference for fatty, calorie-dense foods.

Adverse Events

GI AEs were the most commonly experienced AEs with semaglutide intervention, most specifically nausea and vomiting. Higher rates of GI AEs were observed in patients with lower BMI compared to high BMI. However, these AEs were mostly transient. These trends were also observed in studies with other GLP-1 RAs [24]. Interestingly, weight loss was greater in patients who experienced these AEs compared to those who did not, but these indirect effects led to only a small proportion of total overall weight loss [20]. In the SUSTAIN trials, it was notable that semaglutide had a higher rate of AEs compared to the comparators across all BMI subgroups [20]. Similarly, in this study, there was a high rate of AEs with semaglutide intervention observed, including serious AEs and discontinuations. The shorter-acting GLP-1 RAs along with subcutaneous semaglutide tend to produce the greatest amount of GI AEs compared to longer-acting formulations [27]. As semaglutide’s approved indications and usage have increased, there has been a correlated increase in GI AEs. GI AEs have a dose-dependent relationship and decline over time, with the majority of patients developing these AEs within one week or one month [30,31]. Uptitration for GLP-1 RAs is common, and careful monitoring during the initial phase may help ameliorate these risks. The annual reporting of GI AEs in 2021 has increased almost fourfold compared to those in 2018 [30]. The exact mechanism of GLP-1 RA-associated GI AEs is not fully elucidated but is postulated to be due to central and peripheral GLP-1 receptor activation [30]. A severe and clinically relevant AE that is of great interest is the increased risk of pancreatitis associated with GLP-1 RA usage [30,31]. However, an updated meta-analysis in 2024 by Masson et al. showed no increased risk of pancreatitis across different semaglutide regimens [32]. As the usage of semaglutide and other GLP-1 RAs drastically increases, there may be newer and unexpected AEs that may be reported, especially in the long term. Therefore, due to the high rates of AEs, it may be beneficial to carefully monitor for AEs during the early period of administration, with dose adjustments or supportive treatments as needed to alleviate symptoms and decrease the risk of severe AEs.

Clinical Implications

GLP-1 RAs are a diverse class of agents that vary in terms of molecular composition, pharmacokinetics, pharmacodynamics, and administration modality and frequency [24]. Thus, the individual agents can be analyzed independently and comparative studies would allow a greater understanding of the differences between each agent. Among the plethora of treatment options available, clinicians must take into account comorbidities, cardiovascular benefits, AE risks, impact on weight, cost, and patient preferences to optimize treatment plans and patient outcomes [33]. Therefore, the goal should be assessing the patient’s overall health goal rather than a percentage or numerical weight loss number. In general, patient satisfaction and treatment adherence questionnaires have higher scores with semaglutide versus the other GLP-1 RA comparators [24]. However, this may be attributed to the longer dosing interval of semaglutide, and thus agents that allow once-weekly administration may be favored, leading to increased patient satisfaction.

Limitations

The findings of this study must be contextualized with its limitations. First, the current literature regarding weight loss in type 2 diabetics comparing semaglutide and GLP-1 RAs does not directly compare other available GLP-1 RAs that are not included in this study. This may be a result of unpublished studies or trials that are still ongoing. Second, there is still a relative paucity of trials directly comparing the efficacy and AEs of GLP-1 RAs against each other. The study duration, background treatment before study initiation, and varying dosages among GLP-1 RAs are other important factors to consider. This prevents a definitive statement of the superiority of one GLP-1 RA over another. Future studies should include direct comparative head-to-head studies between GLP-1 RAs along with longer follow-up times to better elucidate the weight loss effects as well as the rate of AEs. Dual agonists (GLP-1/GIP) such as tirzepatide and triple agonists (GLP-1/GIP/glucagon) are also currently in development and investigation. Head-to-head studies with currently FDA-approved GLP-1 RAs versus these novel medications would also help elucidate the benefits of targeting more than one receptor. Third, although this study included seven RCTs, the other two were a nonrandom observational study and a retrospective cohort study. Therefore, the latter two studies may introduce greater bias that can affect the results of the meta-analysis. However, the analysis conducted without the high risk of bias study did not demonstrate significant changes. Fourth, the follow-ups for several studies were short-term and may not accurately capture weight loss and AE outcomes in the long term. Finally, there was notable heterogeneity in the patients included across the studies as well as the dose regimen variants observed.

## Conclusions

GLP-1 RAs have shown efficacy in reducing body weight in diabetic patients with minimal to moderate observed AEs. Semaglutide demonstrated increased numerical weight loss compared to dulaglutide, liraglutide, and exenatide. However, newer dual agonists such as tirzepatide produced a greater weight loss compared to semaglutide. An important consideration is the high rate of AEs which may be further elucidated in the future as the usage of GLP-1 RAs dramatically increases. The paucity of comparative studies prevents a definitive conclusion of the superiority of one GLP-1 RA over another. Additionally, there have been GLP-1 RAs that have recently been developed that act on more than one receptor which may provide even greater weight loss and clinical outcomes for diabetic patients. However, there remains a paucity of literature covering these newer agents. Thus, future studies should compare different GLP-1 RAs to better outline the most optimal and safest formulation of GLP-1 RAs for weight loss.
